# Rapid Increase in SARS-CoV-2 P.1 Lineage Leading to Codominance with B.1.1.7 Lineage, British Columbia, Canada, January–April 2021

**DOI:** 10.3201/eid2711.211190

**Published:** 2021-11

**Authors:** Catherine A. Hogan, Agatha N. Jassem, Hind Sbihi, Yayuk Joffres, John R. Tyson, Kyle Noftall, Marsha Taylor, Tracy Lee, Chris Fjell, Amanda Wilmer, John Galbraith, Marc G. Romney, Bonnie Henry, Mel Krajden, Eleni Galanis, Natalie Prystajecky, Linda M.N. Hoang

**Affiliations:** British Columbia Centre for Disease Control, Vancouver, British Columbia, Canada (C.A. Hogan, A.N. Jassem, H. Sbihi, Y. Joffres, J.R. Tyson, K. Noftall, M. Taylor, T. Lee, C. Fjell, M. Krajden, E. Galanis, N. Prystajecky, L.M.N. Hoang);; University of British Columbia, Vancouver (C.A. Hogan, A.N. Jassem, H. Sbihi, B. Henry, M. Krajden, E. Galanis, N. Prystajecky, L.M.N. Hoang);; Kelowna General Hospital, Kelowna, British Columbia, Canada (A. Wilmer);; Victoria General Hospital, Victoria, British Columbia, Canada (J. Galbraith);; St. Paul’s Hospital, Vancouver (M.G. Romney); Ministry of Health, Victoria (B. Henry)

**Keywords:** variant of concern, testing, public health, COVID-19, coronavirus disease, SARS-CoV-2, severe acute respiratory syndrome coronavirus 2, viruses, respiratory infections, zoonoses, British Columbia, Canada, *Suggested citation for this article*: Hogan CA, Jassem AN, Sbihi H, Joffres Y, Tyson JR, Noftall K, et al. Rapid increase in SARS-CoV-2 P.1 lineage leading to codominance with B.1.1.7 lineage, British Columbia, Canada, January–April 2021. Emerg Infect Dis. 2021 Nov [*date cited*]. https://doi.org/10.3201/eid2711.211190

## Abstract

Several severe acute respiratory syndrome coronavirus 2 variants of concern (VOCs) emerged in late 2020; lineage B.1.1.7 initially dominated globally. However, lineages B.1.351 and P.1 represent potentially greater risk for transmission and immune escape. In British Columbia, Canada, B.1.1.7 and B.1.351 were first identified in December 2020 and P.1 in February 2021. We combined quantitative PCR and whole-genome sequencing to assess relative contribution of VOCs in nearly 67,000 infections during the first 16 weeks of 2021 in British Columbia. B.1.1.7 accounted for <10% of screened or sequenced specimens early on, increasing to >50% by week 8. P.1 accounted for <10% until week 10, increased rapidly to peak at week 12, and by week 13 codominated within 10% of rates of B.1.1.7. B.1.351 was a minority throughout. This rapid expansion of P.1 but suppression of B.1.351 expands our understanding of population-level VOC patterns and might provide clues to fitness determinants for emerging VOCs.

Characterizing mutations in the severe acute respiratory syndrome coronavirus 2 (SARS-CoV-2) genome has led to the identification of variants of concern (VOCs) on the basis of such criteria as increased transmissibility, clinical severity, effect on diagnostic testing, and reduced vaccine efficacy (*1*–*5*). Globally, the B.1.1.7 (Alpha), B.1.351 (Beta), and P.1 (Gamma) lineages represented the 3 main actively circulating VOCs in late 2020 and early 2021 (*6*). B.1.1.7 was first detected in England in September 2020 and progressed to become the dominant lineage in this setting within months (*4*,*7*). By early January 2021, >40 countries had documented B.1.1.7 cases, demonstrating rapid international spread (*8*). This lineage has been associated with an estimated 40%–90% increase in transmissibility (*4*,*7*), variable effects on clinical severity and mortality rates (*5*,*9*,*10*), and limited effect on vaccine effectiveness (*11*). In contrast, whereas B.1.351 and P.1 also emerged in fall 2020 and spread rapidly locally, initial evidence of international transmission beyond South Africa and Brazil was limited (*8*,*12*,*13*). The P.1 lineage poses concern given its associations with an estimated 70%–240% increase in transmissibility (*12*), decreased neutralization capacity by monoclonal and serum-derived polyclonal antibodies (*14*), and increased risk for reinfection (*12*). Limited evidence from Italy, where B.1.1.7 and P.1 lineages have cocirculated, has shown the potential for B.1.1.7 to surpass P.1 for dominant VOC status in a short timeframe (*15*; P. Stefanelli et al., unpub. data, https://www.medrxiv.org/content/10.1101/2021.04.06.21254923v1). However, recent evidence from the United States suggests that infection after vaccination might be attributed to variants characterized by such mutations as E484K, T95I, del142–144, and D614G (*16*). The SARS-CoV-2 spike E484K mutation, which is present in the P.1 and B.1.351 lineages, is most concerning for its potential vaccine response resistance and therefore might theoretically drive selective emergence of these lineages in vaccinated populations (*6*). The factors that lead to the establishment of one strain over another are under study; uncertainty remains regarding the dynamics of VOCs in the context of recent global SARS-CoV-2 vaccine rollout. Understanding the dynamics of VOC rates is critical given the importance of implementing stringent measures to mitigate the spread of more transmissible variants (*17*) and to guide vaccine program development, planning, and delivery.

The province of British Columbia (BC), Canada, population 5.1 million, experienced 3 coronavirus disease (COVID-19) waves during 2020 and early 2021, consistent with other regions in North America and Europe. BC reached a single-day peak of 1,318 cases on April 7, 2021, at the height of the third wave and a cumulative total of 106,985 cases by that point (*18*). For delivery of healthcare services, the province is partitioned into 5 regional health authorities (Appendix Figure 1). B.1.1.7 and B.1.351 lineages were first identified in BC in December 2020 (*19*). BC initiated SARS-CoV-2 vaccination campaigns in December 2020 in predefined phases according to priority populations (*19*). Vaccine administration, which had covered >25% of the population by the end of the study period (epidemiologic week [epiweek] 16), involved three 2-dose vaccines: BNT162b2 mRNA (Pfizer-BioNTech, https://www.pfizer.com), mRNA-1273 (Moderna, https://www.modernatx.com), and ChAdOx1 (AstraZeneca/SII COVISHIELD, https://www.astrazeneca.com) (*19*). The objective of this study was to summarize provincewide VOC surveillance observations over a 16-week period in 2021 spanning epiweek 1 (beginning January 3) to epiweek 16 (beginning April 24), including changes in relative population contribution over time.

## Methods

### VOC Detection by Single-Nucleotide Polymorphism Quantitative PCR and Whole-Genome Sequencing

The British Columbia Centre for Disease Control (BCCDC) Public Health Laboratory (PHL) (Vancouver, BC, Canada) serves as the reference laboratory for the province. In addition, hospital and private laboratories across BC offer frontline SARS-CoV-2 diagnostic testing. Testing using quantitative PCR (qPCR) is largely restricted to symptomatic persons, with the exception of outbreak investigations, which might include asymptomatic testing. We used a combined VOC testing strategy using targeted VOC single-nucleotide polymorphism (SNP) qPCR and whole-genome sequencing (WGS) to monitor VOC prevalence and assessed concordance between the 2 methods. Specimens tested by WGS were from priority populations, such as cases from an outbreak or cluster. Specimens not tested directly by WGS were screened by VOC qPCR. We performed an initial VOC proportion assessment during January 30–February 6, 2021, to evaluate the testing strategy and benchmark VOC prevalence.

### VOC SNP qPCR Implementation

During January 30–March 31, 2021, N501Y qPCR testing was performed at the BCCDC PHL and adopted by the Victoria General Hospital Laboratory (Vancouver Island, BC, Canada). At the same time, St. Paul’s Hospital Virology Laboratory (Vancouver) implemented a sequential qPCR testing algorithm targeting several mutations identified in VOCs, including N501Y and K417T (*20*). The N501Y mutation has been detected in the 3 main currently circulating VOCs: B.1.1.7, B.1.351, and P.1. Among those 3 VOCs, the K417T mutation is found only in P.1. During April 1–24, 2021, VOC qPCR testing was modified to incorporate both N501Y and E484K mutation screening at the BCCDC PHL; this method was adopted by Victoria General Hospital on April 16. Full VOC SNP qPCR used at the BCCDC PHL is described separately (Appendix). This change was performed to account for circulating VOCs and to optimize testing capacity. The E484K mutation has been detected in lineages B.1.351 and P.1 but is very rarely detected in B.1.1.7. In addition, in April 2021, the Kelowna General Hospital Microbiology Laboratory (Kelowna, BC, Canada) implemented a commercially available VOC qPCR targeting N501Y and E484K (Allplex SARS-CoV-2 Variant I Assay; Seegene, https://www.seegene.com). For this study, integrated provincewide surveillance was coordinated by the BCCDC to capture VOC prevalence during January 3–April 24, 2021.

### Confirmation by WGS

Until March 31, 2021, all presumptive positive SNP qPCR results were confirmed by WGS at the BCCDC PHL. After March 31, specimens that tested positive for N501Y alone were identified as presumptive B.1.1.7 lineage; ≈10% were confirmed by WGS. In addition, only ≈25% of specimens that tested positive for N501Y and another mutation were confirmed by WGS. The full WGS methodology performed at the BCCDC PHL is described separately (Appendix).

### Data Linkages and Analysis

We included all cases of SARS-CoV-2 infection diagnosed during January 3–April 24, 2021, for a total of 66,982 cases and 74,057 unique samples. Laboratory data collection was achieved by linking diagnostic SARS-CoV-2 qPCR, VOC SNP qPCR, and WGS databases housed in the BCCDC PHL COVID-19 database. Laboratory sites performing VOC testing provided daily or weekly data transfers of their results to enable the same linkages at the BCCDC PHL. We extracted epidemiologic (demographic and geographic [address of residence] information) and vaccination data (from the Provincial Immunization Registry) on May 15 from the BCCDC Public Health Reporting Data Warehouse and linked that information to laboratory data by using unique personal identifiers shared across the databases. To measure VOC lineage prevalence while better representing community-level dynamics, we did not include VOC lineages that were identified through WGS (as part of cluster investigations or targeted surveillance [e.g., testing after travel]) in this investigation. As part of the WGS testing sample selection, we processed a random selection of samples (background surveillance) by WGS without first conducting SNP qPCR testing. We estimated prevalence of each lineage on the basis of a weighted sum of VOC proportion through each of the 2 pathways of detection, SNP qPCR and WGS (Appendix). We defined a SARS-CoV-2 case as SARS-CoV-2 infection laboratory-confirmed by PCR. To measure VOC proportions among vaccinated case-patients, we defined breakthrough infection as a confirmed SARS-CoV-2 infection reported >21 days after the first (single) vaccine dose and >7 days after the second dose. This definition refers to the number of vaccine doses received across all 2-dose vaccines administered. We calculated descriptive analyses and 95% CI for VOC proportions among vaccinated and unvaccinated persons and for VOC prevalence by using R version 3.5.2 (R Foundation for Statistical Computing, https://www.r-project.org). We used the number of specimens screened by SNP qPCR (N501Y/E484K duplex or sequential qPCR algorithm) or background WGS as the denominator. We used Kruskal-Wallis analysis for comparison of continuous variables and the Fisher exact test for categorical variables. This work was conducted under the public health mandate, and institutional review board approval was waived.

## Results

During the study period, 66,982 cases of SARS-CoV-2 infection were identified in BC, of which 19,768 (31.9%) were identified as infections with a VOC. Most VOC case-patients were young adults, median age was 33 (range <1 to 99) years, and sex distribution was approximately equal (52.2% male) (Appendix Table). Age and sex distribution varied significantly by VOC, however; P.1 case-patients were younger and more likely to be men than case-patients who tested positive for the other 2 VOCs.

**Figure 1:**
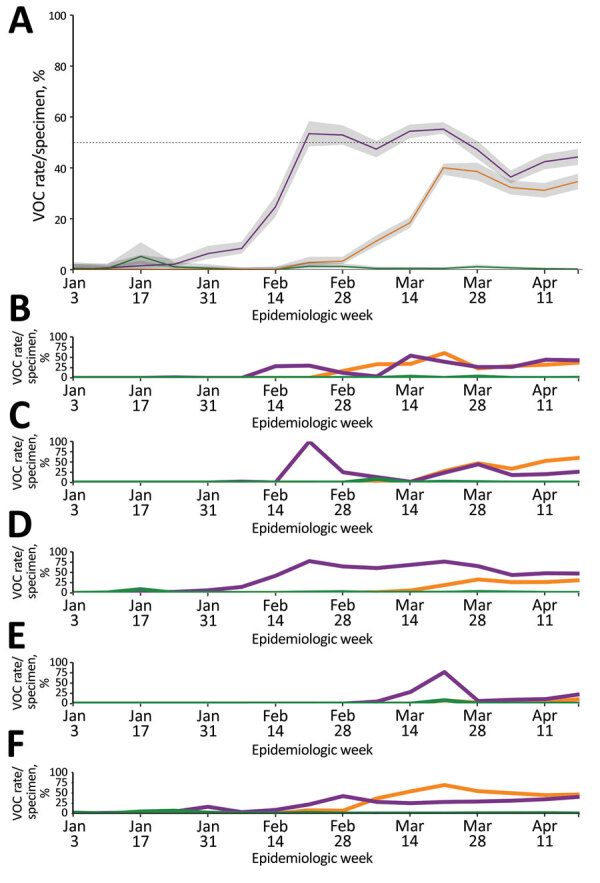
Weekly rate estimates of each severe acute respiratory syndrome coronavirus 2 VOC (per 100 specimens screened or sequenced), by epidemiologic week and specimen collection date, British Columbia (BC), Canada, January–April 2021. The 3 main VOC are shown in purple (B.1.1.7), green (B.1.351), and orange (P.1). The P.1 lineage was confirmed through whole-genome sequencing or from an N501Y- and E484K-positive or K417T-positive result from epiweek 12 onward. A) VOC data for the whole province. Shaded areas around the line represent 95% CI; dashed line indicates 50%. B) VOC data for BC regional health authority 1. C) VOC data for BC regional health authority 2. D) VOC data for BC regional health authority 3. E) VOC data for BC regional health authority 4. F) VOC data for BC regional health authority 5. The 95% CIs are not shown for health regions because of low numbers and rates and the resulting wide uncertainty seen across regions for extended periods. BR, Brazil; SA, South Africa; UK, United Kingdom; VOC, variant of concern.

During the initial BC VOC prevalence assessment, 3,024 specimens were tested during January 30–February 6, 2021, representing 97.5% of all laboratory-confirmed SARS-CoV-2–positive specimens in the province. Just 28 (0.93%) of these 3,024 SARS-CoV-2–positive specimens were identified as VOCs. Of those identified as VOCs, 22/28 (79%) were identified through screening qPCR and 6/28 (21%) through direct WGS. Of the 22 qPCR-screened specimens, 21 were successfully sequenced; the qPCR VOC confirmation rate by WGS was 95.5%, reinforcing the value of the VOC qPCR as a screening strategy. VOC cases were characterized as 23 (85.2%) B.1.1.7 lineage and 4 (14.8%) B.1.351 lineage. Continued surveillance by VOC screening of nearly all SARS-CoV-2–positive specimens identified through diagnostic testing showed a progressive increase in overall VOC-positivity in BC, reaching >10% by the end of February 2021, >50% by the end of March 2021, and >70% by mid-April 2021 (Table). By VOC case count, the B.1.1.7 lineage increased progressively from 0% to 7.9% during epiweeks 1–6, then increased more rapidly to 52.2% during epiweeks 6–8 (Figure 1, panel A); estimated doubling rate was <1 week. The P.1 lineage was initially recognized in BC at the end of February 2021, and rapidly increased to account for 39.4% of VOCs by epiweek 12; the minimal estimated doubling time was <1 week during epiweeks 10–12 (Figure 1, panel A). By epiweek 14, the proportion of B.1.1.7 and P.1 was similar, ranging from 32.3%–36.5%, and both stabilized. This rapid P.1 increase was clearly observed in 3 regional health authorities in BC (regions 1, 2, and 5); B.1.1.7 was initially predominant (Figure 1, panels B, C, F). In the 2 other BC health regions (regions 3 and 4) (Figure 1, panels D, E), P.1 increased modestly overall and did not compete with B.1.1.7 as the dominant lineage. However, when we restricted the analysis to a single smaller geographic unit of region 3 in which B.1.1.7 had been circulating for >8 weeks, we observed a rapid increase in P.1, after which the 2 lineages coexisted (Appendix Figure 2). Despite earlier detection of B.1.351 in BC in epiweek 9, B.1.351 remained stable or decreased over time and represented <10% of all VOC cases across the entire study (Figure 1, panel A).

**Table:** Number of specimens positive for severe acute respiratory syndrome coronavirus 2, proportion screened by variant of concern assay, and proportion positive for variants of concern, British Columbia, Canada, January 3–April 24, 2021*

Epiweek	Start date	No. positive specimens	No. (%) specimens	
			Screened by VOC assay	Presumptive VOC-positive†
1	2021 Jan 3	3,857	19 (0.49)	0
2	2021 Jan 10	3,498	235 (6.72)	2 (0.85)
3	2021 Jan 17	3,477	867 (24.94)	9 (1.04)
4	2021 Jan 24	3,325	793 (23.85)	8 (1.01)
5	2021 Jan 31	3,125	2,200 (70.40)	24 (1.09)
6	2021 Feb 7	3,126	2,263 (72.39)	57 (2.52)
7	2021 Feb 14	3,464	2,821 (81.44)	105 (3.72)
8	2021 Feb 21	3,638	3,291 (90.46)	231 (7.02)
9	2021 Feb 28	3,867	3,813 (98.86)	442 (11.59)
10	2021 Mar 7	3,862	3,862 (100)	626 (16.20)
11	2021 Mar 14	4,155	4,128 (99.35)	1,081 (26.19)
12	2021 Mar 21	5,723	5,636 (98.48)	2,162 (38.36)
13	2021 Mar 28	7,036	7,032 (99.94)	3,622 (51.51)
14	2021 Apr 4	8,195	8,185 (99.88)	5,404 (66.02)
15	2021 Apr 11	7,278	6,560 (90.13)	4,644 (70.79)
16	2021 Apr 18	6,441	6,127 (95.12)	4,681 (76.40)

*Epiweek, epidemiologic week; VOC, variant of concern. †Presumptive VOC detection refers to VOC identification by quantitative PCR testing without confirmation by whole-genome sequencing; includes all specimens tested for VOCs.

**Figure 2:**
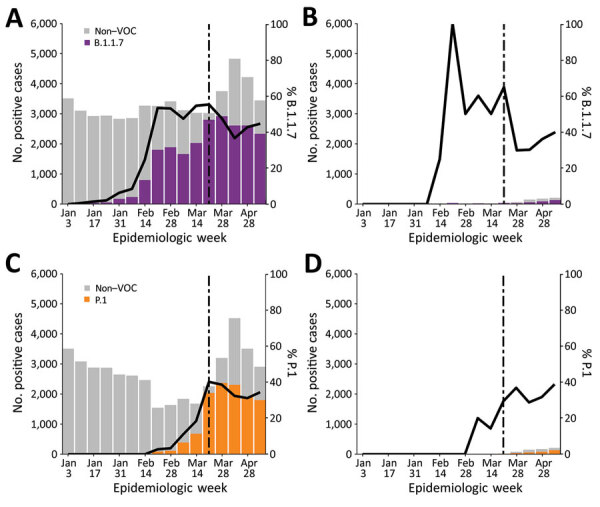
Distribution of all severe acute respiratory syndrome coronavirus 2 cases and VOC cases by vaccination status, British Columbia, Canada, January–April 2021. Vaccinated persons included those who had received 1 dose of a 2-dose vaccine with diagnosis confirmed by PCR >21 days after the first dose (on the basis of specimen collection date). Stacked bars (left-hand y-axis) represent the absolute number of VOC cases and non-VOC cases. Solid lines (right-hand y-axis) show the percentage of VOC among all cases identified in a given week. Dotted lines show the date when VOC quantitative PCR (qPCR) including E484K mutation detection was adopted at the British Columbia Centre for Disease Control Public Health Laboratory. A) B.1.1.7 and non-VOC cases in nonvaccinated persons. B) B.1.1.7 and non-VOC cases in vaccinated persons. VOC cases in panels A and B included B.1.1.7 confirmed for lineage by whole-genome sequencing and presumptive B.1.1.7 positives based on a VOC qPCR result of N501Y-positive and E484K-negative. C) P.1 and non-VOC cases in nonvaccinated persons. D) P.1 and non-VOC cases in vaccinated persons. VOC cases in panels C and D included P.1 and B.1.351 confirmed for lineage by whole-genome sequencing and presumptive P.1 positives based on a VOC qPCR result of N501Y-positive and E484K-positive or K417T-positive. VOC, variant of concern.

During this study period, 1,280 breakthrough infections were identified. Among those, 497 (1.7%) cases in persons who had received 1 vaccine dose were attributed to B.1.1.7 and P.1 lineage strains, and 18 (0.2%) cases in persons who had received 2 doses were attributed to B.1.1.7 and P.1 lineage strains (Appendix Table). Infections after 2 doses of vaccine were excluded from downstream analyses given their small number. Almost all (96.4%) of the VOC infections occurred in unvaccinated persons, but approximately the same proportion of VOC cases occurred among partially vaccinated and unvaccinated persons. Specifically, during epiweeks 9–16, when B.1.1.7 was widespread and case counts were high, B.1.1.7 infections were identified in 37%–55% of cases in unvaccinated persons and in 30%–65% of cases in persons who had received 1 dose (Figure 2). During epiweeks 10–16, after P.1 emerged in the study population, the proportion of infections with P.1 was 14%–39% among cases in partially vaccinated persons and 11%–40% among cases in unvaccinated persons (Figure 2). Conversely, at the same time (epiweeks 9–16), 6%–50% of breakthrough infections were non-VOC lineages in persons who had received 1 dose of a 2-dose vaccine.

## Discussion

Results from this analysis of VOC laboratory and epidemiologic surveillance data demonstrated initially low prevalence of VOC and predominance of the B.1.1.7 lineage in BC, Canada, in early 2021, consistent with trends documented across North America. An earlier study that tested 2,618 SARS-CoV-2–positive samples in BC over a 7-day period in a single regional health authority reported an outbreak of 13 P.1 cases; however, whether this occurrence represented a single confined outbreak or potential for more disseminated spread of this lineage is uncertain, and WGS data were limited (*20*). Building on those earlier findings, our study performed ongoing surveillance of >74,000 SARS-CoV-2–positive specimens across the entire province over 16 weeks. This surveillance led to the detection of a rapid and substantial increase in P.1 lineage, demonstrating its potential for codominance with B.1.1.7 at the provincial level. The pattern of population-level lineage change over time reflected the largest outbreak of the P.1 lineage outside of Brazil at that time (*21*,*22*). This study documented the parallel rapid increase of the P.1 lineage in 3 regional health authorities in which B.1.1.7 was previously established, contrasting with previous reports in Italy showing sustained dominance of B.1.1.7 after the introduction of P.1 (*15*; P. Stefanelli et al., unpub. data). In 2 regions, the proportion of P.1 exceeded that of B.1.1.7 for a sustained period. Of note, P.1 arose to codominance before broad vaccination of the most likely implicated young adult age group, and the proportion of VOCs was similar between vaccinated and unvaccinated groups, suggesting that vaccination was not driving the observed trends of P.1 increase. Although our findings contradict those of Hacisuleyman et al. (*16*), which cautioned that infections after vaccination might be characterized by variant mutations such as E484K, the difference might reflect the small sample size in that study. Comprehensive comparative demographic data to characterize the P.1 lineage are lacking; however, early data from Brazil demonstrate increased case-fatality rates among younger age groups that coincide temporally with the rise of this lineage there (M.H.S. de Oliveira, unpub. data, https://www.medrxiv.org/content/10.1101/2021.03.24.21254046v1). Further work investigating the full epidemiologic characteristics and clinical implications, including disease severity, of the P.1 increase will complement the findings of this study.

The first limitation of our study is that the VOC qPCR and WGS confirmation testing strategies were modified over time, which might partially limit comparability of positivity estimates over time and could overestimate rates of P.1 because of the use of E484K-positivity as its surrogate in some instances. Nonetheless, background surveillance data during the same timeframe (data not shown) supported the identification of most E484K-positive specimens as P.1 lineage. Second, to avoid oversampling bias, we based the WGS selection strategy on the inclusion of specimens from persons tested for background surveillance purposes, not for outbreak investigation or targeted (e.g., travel-related) surveillance. Third, the populations that were vaccinated during this study period do not necessarily reflect the persons at highest risk for VOC infection, which might have modified breakthrough VOC proportions. Of note, this study was not designed to assess vaccine effectiveness; we did not adjust for confounders in the relationship between vaccination and infection, such as age, underlying conditions, vaccination program roll-out, and temporal-spatial epidemic risk. More comprehensive studies considering characteristics of the vaccine roll-out strategy are needed for analyses beyond overall comparisons between lineages in unvaccinated and vaccinated groups.

In summary, this population-level study based on a combined qPCR and WGS VOC testing strategy demonstrated the rapid increase of the P.1 lineage and its later codominance, contrasting with studies in settings such as Italy, where the B.1.1.7 and P.1 lineages have cocirculated. Further work is required to elucidate the biologic and social factors that enabled the establishment of this lineage and to assess the clinical implications of these findings.

Appendix Additional information about rapid increase in SARS-CoV-2 P.1 lineage leading to codominance with B.1.1.7 lineage, British Columbia, Canada, January–April 2021
