# Supplementary material for: Rapid Increase in SARS-CoV-2 P.1 Lineage Leading to Codominance with B.1.1.7 Lineage, British Columbia, Canada, January–April 2021
Source: Emerg Infect Dis. 2021 Nov;27(11):2802–9. doi: 10.3201/eid2711.211190 (PMC8544957; doi:10.3201/eid2711.211190)
Supplement: Appendix — Additional information about rapid increase in SARS-CoV-2 P.1 lineage leading to codominance with B.1.1.7 lineage, British Columbia, Canada, January–April 2021 [file 21-1190-Techapp-s1.pdf]

Article DOI: https://doi.org/10.3201/eid2711.211190

Rapid Increase in SARS-CoV-2 P.1 Lineage Leading to Codominance with B.1.1.7 Lineage, British Columbia, Canada, January–April 2021

Appendix

Methods

N501Y and E484K Mutation Quantitative PCR Screening

The British Columbia Centre for Disease Control Public Health Laboratory developed a real-time, multiplex reverse transcription PCR (RT-PCR) assay targeting the envelope (E) gene and the N501Y mutation of the spike (S) protein, with later addition of the S protein E484K mutation. The E gene probe was used to confirm detection of severe acute respiratory syndrome coronavirus 2 (SARS-CoV-2) RNA in the specimen, as previously described by Corman et al. (*1*). Primer and probe details for variant of concern (VoC) detection are as follows: for N501Y, F-AATTGTTACTTTCCTTTACAATCATATGG, R-CCACAAACAGTTGCTGGTGC, FAM-MGB-Probe-ACACTAGCCATCCTTACTGCGCTTCG; for E484K, F-AGAGAGATATTTCAACTGAAATCTATCAGG, R-CCACAAACAGTTGCTGGTGC, MGB-Probe-CTTGTAATGGTGTTAAAGGT. In brief, total nucleic acids were extracted from 200µL of upper respiratory specimen matrix (universal transport medium or saline) by using the Applied BioSystems MagMax Express 96 Nucleic Acid Extractor and the MagMax Viral/Pathogen Nucleic Acid Isolation Kit (ThermoFisher Scientific, https://www.thermofisher.com) according to the manufacturer's recommendations. Real-time RT-PCR was performed by using the TaqMan Fast Virus Master Mix (ThermoFisher Scientific) on the Applied Biosystems 7500 FAST real-time PCR system (ThermoFisher Scientific). PCR set-up was performed by using a volume of 5µL of patient specimen extract, for a final reaction volume of 20µL.

Whole-Genome Sequencing

Whole-genome sequencing was performed at the British Columbia Centre for Disease Control Public Health Laboratory. In brief, SARS-CoV-2 RNA was extracted by using the Applied BioSystems MagMax Express 96 Nucleic Acid Extractor and the MagMax Viral/Pathogen Nucleic Acid Isolation Kit (ThermoFisher). Viral RNA was reverse transcribed into cDNA by using Thermo SuperScript IV, and a 2-pool multiplex PCR with primers tiled across the entire SARS-CoV-2 genome in 29 ≈1200bp segments was performed (*2*). We prepared DNA libraries for whole-genome sequencing on a MiSeq or NextSeq 2000 instrument (Illumina, https://www.illumina.com) by using DNA Prep Library Preparation Kit (Illumina). SARS-CoV-2 whole-genome consensus sequences and mutation compositions were generated by using a modified Nextflow pipeline for running the ARTIC network (https://artic.network/ncov-2019) fieldbioinformatics tools (https://github.com/BCCDC-PHL/ncov2019-artic-nf). Reports detailing SARS-CoV-2 lineage information, sequencing QC metrics, and mutational profiles were generated by using ncov-tools from the Simpson Lab (https://github.com/jts/ncov-tools).

Weighting Procedure for VoC Proportion

Where Ws is the weight for sequencing and Wg is the weight for genomics:

Ws = total screened / (total screened + total sequenced)

Wg = total sequenced / (total screened + total sequenced)

For a given VoC called i (where i could be B.1.1.7, B.1.351, or P.1 lineage)

Prevalence (i) = [presumptive positive (i) / (total screened)] × Ws + [sequenced (i) / total sequenced] × Wg

References

1. Corman VM, Landt O, Kaiser M, Molenkamp R, Meijer A, Chu DK, et al. Detection of 2019 novel coronavirus (2019-nCoV) by real-time RT-PCR. [Erratum in: Euro Surveill. 2020;25(14):20200409c; 2020;25(3):2007303; 2021;26(5):210204e]. Euro Surveill. 2020;25:1–8. PubMed https://doi.org/10.2807/1560-7917.ES.2020.25.3.2000045

2. Freed NE, Vlkova M, Faisal MB, Silander OK. Rapid and inexpensive whole-genome sequencing of SARS-CoV-2 using 1200 bp tiled amplicons and Oxford Nanopore Rapid Barcoding. Biol Methods Protoc. 2020;5:bpaa014.

3. Government of British Columbia. Regional health authorities. 2021 [cited 2021 Jul 14]. https://www2.gov.bc.ca/gov/content/health/about-bc-s-health-care-system/partners/health-authorities/regional-health-authorities

Appendix Table. Descriptive demographics and vaccine information of persons infected with severe acute respiratory syndrome coronavirus 2 and included in the study by the 3 main variants of concern, British Columbia, Canada, January–April, 2021*

No. persons	Non-VoC† 47,159 (70.5)	B.1.1.7 11,614 (17.4)	B.1.351 75 (0.1)	P.1 8,079 (12.1)	p value (VoCs)‡
Sex					0.0041
F	22,667 (48.1)	5,637 (48.5)	36 (48)	3,756 (46.5)	
M	24,442 (51.8)	5,960 (51.3)	38 (50.7)	4,312 (53.4)	
Vaccination status					0.0073
Not vaccinated	46,394 (98.4)	11340 (97.6)	75 (100)	7,838 (97)	
1 dose	668 (1.4)	260 (2.2)		237 (2.9)	
2 doses	97 (0.2)	14 (0.1)		4 (0)	
Vaccine type					0.45
AstraZeneca	49 (0.1)	26 (0.2)		26 (0.3%)	
Moderna mRNA-1273	88 (0.2)	16 (0.1)		20 (0.2%)	
Pfizer mRNA BNT162b2	628 (1.3)	232 (2)		195 (2.4%)	
Age, y					<0.001
Median (range)	35 (0–108)	34 (0–99)	38 (1–83)	32 (0–96)	
IQR	23–51	22–49	25–58	22–46	
Time from sample collection to vaccine, d					0.29
Median (range)	35 (19–124)	36 (18–118)	NA	33 (19–106)	
IQR	26–54	26–53.8	NA	26–49	

*Values are no. (%) except as indicated. Missing data for each variable are excluded. IQR, interquartile range; NA, not applicable; VoC, variant of concern.
†Non-VoC represents, in effect, samples not screened or sequenced and is therefore an estimate. Although we screened >95% of all positive tests, it is expected that a negligible proportion of positive samples were not captured, and B.1.351 reflects only sequencing results given the assumption that dual positive results of VoC quantitative PCR were highly likely to be P1.
‡p values represent comparison among 3 variants of concern and were calculated by Kruskal-Wallis test for continuous variables and Fisher exact test for categorical variables.

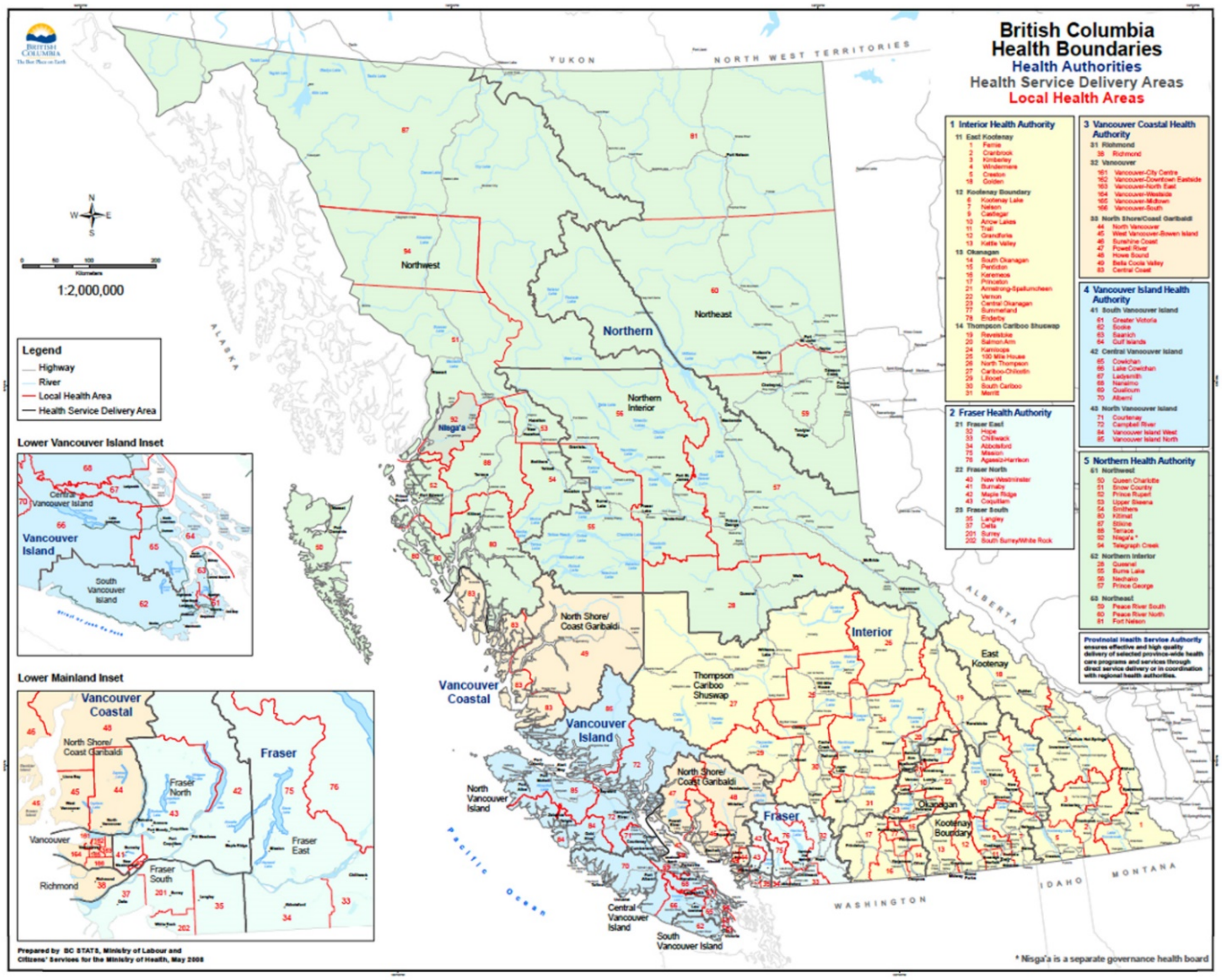

Appendix Figure 1. Map of the 5 regional health authorities, British Columbia, Canada (*3*).

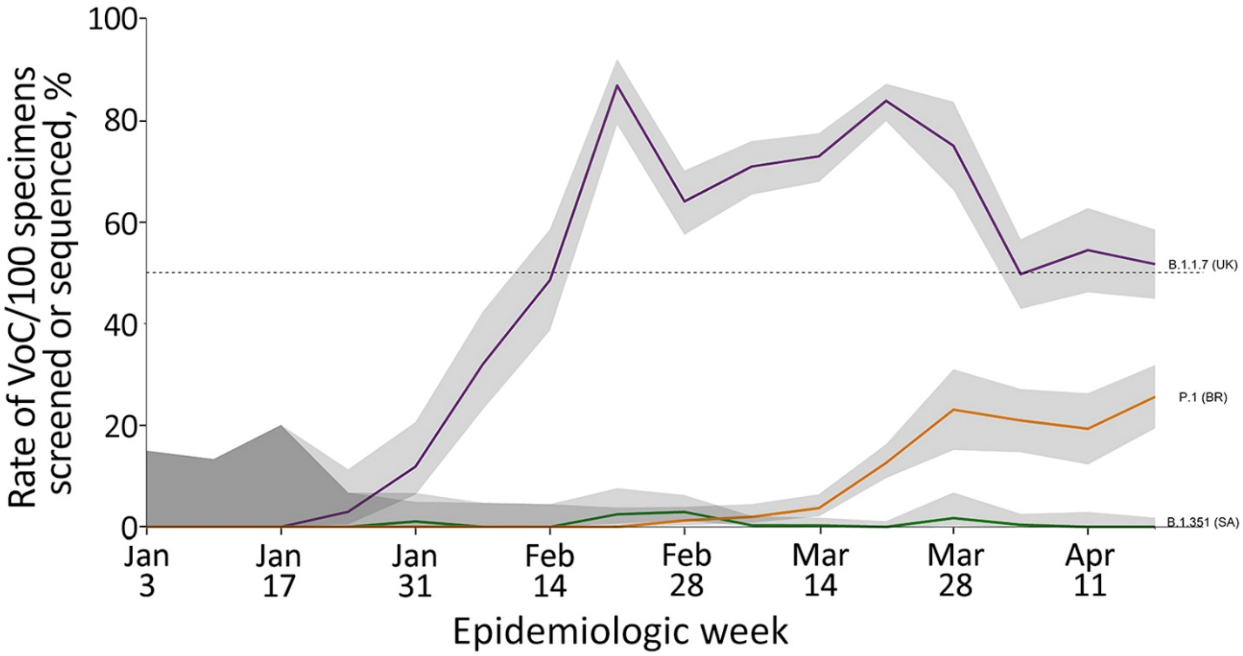

Appendix Figure 2. Weekly rate estimate of each VoC/100 specimens screened or sequenced, by epiweek and specimen collection date, for a single smaller geographic unit within a regional health authority, British Columbia, Canada. The 3 main VoCs are shown in purple (B.1.1.7), green (B.1.351), and orange (P.1). The dashed line indicates 50%. The P.1 lineage was identified through whole-genome sequencing confirmation or from an N501Y-positive and E484K-positive or K417T-positive result from epiweek 12 onward. Shaded areas around the line represent 95% CI. VoC, variant of concern.
